# KSHV Latency Locus Cooperates with Myc to Drive Lymphoma in Mice

**DOI:** 10.1371/journal.ppat.1005135

**Published:** 2015-09-01

**Authors:** Sang-Hoon Sin, Yongbaek Kim, Anthony Eason, Dirk P. Dittmer

**Affiliations:** 1 Department of Microbiology and Immunology, Program in Global Oncology, Lineberger Comprehensive Cancer Center, and Center for AIDS Research, The University of North Carolina at Chapel Hill, Chapel Hill, North Carolina, United States of America; 2 Department of Veterinary Medicine, College of Veterinary Medicine, Seoul National University, Seoul, South Korea; University of Southern California, UNITED STATES

## Abstract

Kaposi sarcoma-associated herpesvirus (KSHV) has been linked to Kaposi sarcoma and B-cell malignancies. Mechanisms of KSHV-induced oncogenesis remain elusive, however, in part due to lack of reliable in vivo models. Recently, we showed that transgenic mice expressing the KSHV latent genes, including all viral microRNAs, developed splenic B cell hyperplasia with 100% penetrance, but only a fraction converted to B cell lymphomas, suggesting that cooperative oncogenic events were missing. Myc was chosen as a possible candidate, because Myc is deregulated in many B cell lymphomas. We crossed KSHV latency locus transgenic (latency) mice to Cα Myc transgenic (Myc) mice. By itself these Myc transgenic mice develop lymphomas only rarely. In the double transgenic mice (Myc/latency) we observed plasmacytosis, severe extramedullary hematopoiesis in spleen and liver, and increased proliferation of splenocytes. Myc/latency mice developed frank lymphoma at a higher rate than single transgenic latency or Myc mice. These data indicate that the KSHV latency locus cooperates with the deregulated Myc pathways to further lymphoma progression.

## Author Summary

Kaposi’s sarcoma-associated herpesvirus (KSHV) is associated with Kaposi sarcoma as well as the B-cell malignancies primary effusion lymphoma (PEL) and multicentric Castleman’s disease (MCD). Only a few KSHV genes, including all micro RNAs, are expressed in latent infection of B cells. We already showed that KSHV latency locus transgenic mice consistently develop B cell hyperplasia. To find out possible host contributions to lymphomagenesis we evaluated the Myc oncogene. Compound KSHV latency locus and Myc mice developed plasmacytosis exemplified by increased frequency of plasma cells in the spleen, a high accelerated lymphoma development, and severe extramedullary hematopoiesis. These data show that the KSHV latency locus can cooperate with Myc activation in viral lymphomagenesis.

## Introduction

Myc encodes a multifunctional protein which is involved in many biological functions, including transcriptional control, cell cycle, signal transduction, oncogenesis, and development (reviewed in [1]). Recurrent deregulation of c-Myc (Myc) is a hallmark of many lymphoma such as Burkitt lymphoma (BL) and a fraction (~20%) of diffuse large B cell lymphomas (DLBCL), including post-germinal center (GC), non-Hodgkin’s lymphoma [1,2]. The most frequent chromosomal translocation is t(8;14)(q24;q32) found in BL, which relocates Myc from 8q24 to the immunoglobulin heavy chain (IgH) locus on 14q32. Some cases of DLBCL, such as anaplastic lymphoma kinase (ALK) positive large B-cell lymphoma do not carry Myc translocation per se, but overexpress Myc protein [3,4]. This suggests that deregulated expression of the Myc protein by any means contributes to B cell lymphomagenesis.

Over the years, multiple mouse models of Myc-driven lymphomas have been developed [5–12]. The first and most aggressive transgenic model used the mouse Myc gene, driven by the IgH μ enhancer (EμMyc mouse); here the transgene induced tumors, expansion of lymph nodes, and lymphoid malignancy within 6–15 weeks [5]. Transgenic mice expressing a translocated Myc gene from a human BL cell line under the Igλ light chain regulatory sequences also readily developed lymphomas [8], whereas transgenic mice with a specific, single copy targeted insertion into the Cα of the IgH locus (iMycCα mouse), which mimic the t(8;14) in BL, developed B cell lymphomas with very low incidence [7]. In sum, the phenotypes of Myc mouse models range from moderate to fully penetrant, aggressive lymphomagenesis depending on the particulars of the transgene regulatory context, each mimicking different types and/or stages of lymphomagenesis.

Using these mouse models, many factors were uncovered that cooperate with Myc. Targeted overexpression of N-ras in B cells promoted B cell neoplasia in conjunction with Myc [13]. There is also evidence for cooperation of interleukin-6 (IL-6) with Myc in plasma cell tumor development [14]. Furthermore, B cell receptor (BCR) activation was shown to promote B cell lymphomagenesis in conjunction with Myc [15]; and using a CD19 knockout mouse model, the CD19 signaling loop was revealed to promote development and progression of B cell lymphoma [16]. CD19 is an essential accessory to the BCR signaling leading to phosphoinositide-3-kinase (PI3K) activation [17]. Myc itself was shown to synergize with PI3K signaling to provoke BL [18].

Kaposi sarcoma-associated herpesvirus (KSHV) is an oncogenic human γ-herpesvirus. KSHV is implicated in the pathogenesis of Kaposi sarcoma, primary effusion lymphoma (PEL), multicentric Castleman’s disease (MCD), and some instances of DLBCL (reviewed in [19]). Whereas MCD is a pre-malignant, relapsing-remitting-type GC hyperplasia, PEL is a highly aggressive post-GC DLBCL. An association between KSHV and microlymphoma has been suggested as well [20,21]. Typically KSHV persists in the B cell compartment for many years prior to overtly symptomatic MCD or lymphoma.

Latency is the default replicative pathway of KSHV in B cells (reviewed in [22]). Only very few of the more than 80 viral genes are expressed [23,24]. Those, which are consistently detectable in every single infected B cell, include the latency-associated nuclear antigen (LANA), a viral homolog of cellular cyclin D2 (vCYC), a viral FLICE inhibitory protein (vFLIP), K12 (kaposin), all viral micro RNAs (miRNAs), and v-IRF3/LANA-2 [24,25]. Many of these genes have been implicated in B cell signaling in tissue culture, but only few have been explored in vivo. This represents a gap in our understanding and a barrier towards pre-clinical testing of targeted anti-KSHV lymphoma agents.

Expression of LANA alone in B cells resulted in hyperplasia, low-penetrance lymphoma, and drastically increased BCR responses to a T cell-dependent (TD) antigen. Analogous to the transgenic Myc models, this phenotype was dependent on CD19 [26–28]. Mice expressing the entire KSHV latency locus, including all viral miRNAs, in a pure C57BL/6 background exhibited even more increased BCR responses to TD antigen, and also displayed marginal zone (MZ) enlargement, as well as plasmacytosis and frank lymphoma [29]. Whereas these KSHV latency mice exhibited GC and MZ hyperactivity akin to MCD with 100% penetrance and at a normal age, long latency was needed for lymphoma development with incomplete penetrance. This suggested that additional, cellular driver events would accelerate lymphomagenesis. Recent studies suggested that Myc is frequently deregulated by KSHV latent proteins such as LANA and vIRF3 [30–32]. Though structural abnormalities involving Myc translocations are not seen in PEL [30,33], this does not mean that Myc couldn’t be activated at the transcriptional and post-transcriptional level either by viral or cellular events.

To test the hypothesis that Myc was one of the host factors, which can augment KSHV-driven B cell lymphomagenesis, we utilized transgenic mice carrying the very weak IgH Cα Myc allele. As mentioned above this particular Myc allele on its own induced hyperplasia, but not lymphoma [7]. We found that the KSHV latent genes synergized with Myc to drive lymphoma in vivo.

## Results

### Generation of double transgenic, Myc/latency mouse line

We had previously reported the KSHV latency locus transgenic mouse line, which expresses the KSHV latent genes and all miRNAs in B cells, albeit at low levels [29]. In the latency mice, the MZ and plasma cell frequencies were increased and frank tumors developed (~16% / 300 days) [29]. We chose Myc transgenic mice, where a Histidine-tagged Myc coding region was inserted into IgH Cα locus under its own promoter and Eα enhancer to mimic the Myc-activating chromosomal translocation t(12;15). T(12;15) defines 90% of plasma cell tumors found in pristane-treated BALB/c mice [7,34]. These plasmacytomas develop as liquid ascites in the body cavities of the animal and represent a phenotype of mouse lymphoma closely resembling human PEL. However, the tumor incidence rate of the iMycCα single transgenic mice was low and lymphoma developed only after a long latency period (~9%/ 300 days) [7]. This made them ideal to uncover synergy between host Myc and KSHV latent genes.

To study the cooperative interaction between the KSHV latency locus and Myc in viral lymphomagenesis, the latency mice were crossed to iMycCα mice to generate a double-transgenic mouse line, which expresses the KSHV latency locus in the context of activated Myc, termed Myc/latency. Genotyping for the KSHV transgene was done as previously described [29]. The presence of the Myc transgene was confirmed by allele specific PCR (S1 Fig). We confirmed that the KSHV miRNAs and mRNAs of KSHV latent genes were expressed in the presence of the Myc transgene similarly as in the latency mice line (S2 Fig; see also reference [29]).

### Higher plasma cell frequency in the Myc/latency mice

**Fig 1:**
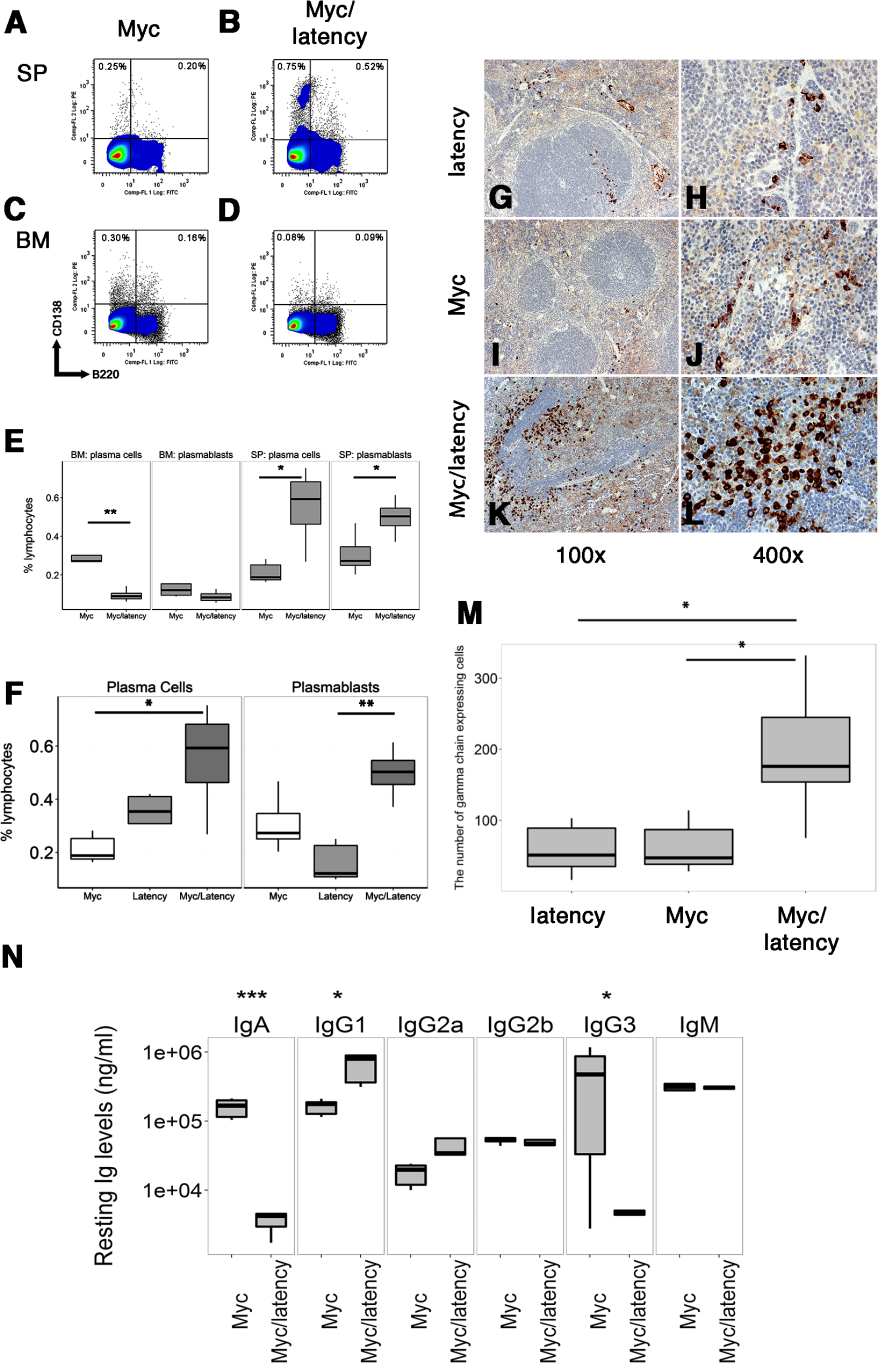
Increased frequency of PCs in Myc/latency mice. Cells were isolated from spleen or BM from 7–11 week-old Myc (n = 5) or the Myc/latency (n = 5) mice and analyzed using flow cytometry. Lymphocytes in spleen or BM were pregated based on CD19 expression. CD19^-^ cells were further gated using CD138 and B220. Representative FACS plots of PBs and PCs were shown. **(A-B)** Splenic PBs (CD19^-^B220^+^CD138^+^) and PCs (CD19^-^B220^-^CD138^+^). **(C-D)** PBs and PCs in BM. **(E)** The percentages of PBs or PCs are shown. Splenic plasmacytosis induced by increased PCs was further confirmed by immunostaining with γ heavy-chain. **(F)** Comparison of splenic PBs and PCs frequencies from the latency (n = 5), Myc (n = 5), and Myc/latency (n = 5) mice. Splenic cells were isolated from 7–11 week-old mice and analyzed by flow cytometry. Igγ chain staining was performed for spleen sections from the latency mouse **(n = 5; G-H)**, single transgenic Myc mouse **(n = 5; I-J)**, and double transgenic Myc/latency mouse **(n = 5; K-L)**. Representative images were shown. **(M)** The number of Igγ chain positive cells from 400X images (n = 5 for all genotypes) was counted and plotted. **(N)** Isotype-specific Ig regulation by KSHV latency locus in overexpressed Myc background. Levels of Igs were measured by ELISA and plotted from the Myc mice (n = 6), and Myc/latency (n = 5). *, **, and *** represent significant difference with p ≤ 0.05, p ≤ 0.005, p ≤ 0.0005 by ANOVA, respectively.

The KSHV latency locus alone induced plasmacytosis [29], and this phenotype was maintained in the compound Myc/latency mice, though other phenotypes of original latency mice, such as increased frequency of mature and MZ B cells, were not recapitulated in the Myc/latency mice (S1 Table). Plasmablasts (PBs; CD19^-^B220^+^CD138^+^) and plasma cells (PCs; CD19^-^B220^-^CD138^+^) were increased in the spleens of Myc/latency mice compared to Myc mice (Fig 1A and 1B). This increase was statistically significant to p ≤ 0.03 by ANOVA (Fig 1E). The increased numbers of PCs were confirmed in situ using Igγ chain immunohistochemistry. The intensity and prevalence of the staining was more robust in spleen sections of Myc/latency mice compared to those of Myc single transgenic mice (Fig 1G–1M). This phenotype was consistently seen in all mice (S3 Fig). Next, the frequencies of PBs and PCs in Myc/latency mice were compared to those of the latency mice. PBs were increased significantly in the Myc/latency mice compared to the latency mice (p ≤ 0. 001 by ANOVA), while PCs were not augmented obviously (p ≤ 0.08 by ANOVA, Fig 1F). The direct comparison of splenic PBs and PCs from the latency, Myc, and Myc/latency mice strongly suggests that the additive effect of KSHV latency locus and Myc overexpression induces higher PC and PB frequencies (Fig 1F). These data demonstrate that increased frequency of PCs in the Myc/latency mice is not a single effect of the KSHV latency locus, but the result of cooperation between the KSHV transgene and the Myc transgene. Thus, activated Myc may cooperate with KSHV latent genes to drive plasma cell proliferation/activation.

A similarly increased frequency of PBs was not observed in bone marrow (BM) (Fig 1C and 1D) rather, PCs in BM of the Myc/latency mice was considerably decreased compared to that of Myc mice (Fig 1E; p ≤ 0.002 by ANOVA). This suggests that the KSHV latency locus induces PBs, short-lived PCs, and some long-lived PCs in GC of the spleen, but interferes with homing of long-lived PCs and migratory PBs to BM in the presence of deregulated Myc.

We observed elevated peripheral blood IgG_1_ levels, while IgA and IgG_3_ levels were decreased in the Myc/latency, compared to the Myc mice (Fig 1N). The elevation was consistent and pronounced enough in the absence of any specific antigenic stimuli to be diagnosed as hyperglobulinemia. KSHV latency transgenic mice alone also displayed hyperglobulinemia of IgG_1_, IgG_3_, and IgM [24], while no significant difference in Ig levels has been reported for Myc mice compared to wild-type mice [7]. As before [29], the phenotype of the KSHV latency locus manifested itself in the context of forced Myc expression.

### Increased proliferation, GC formation and antigen responsiveness in Myc/latency compound transgenic mice

**Fig 2:**
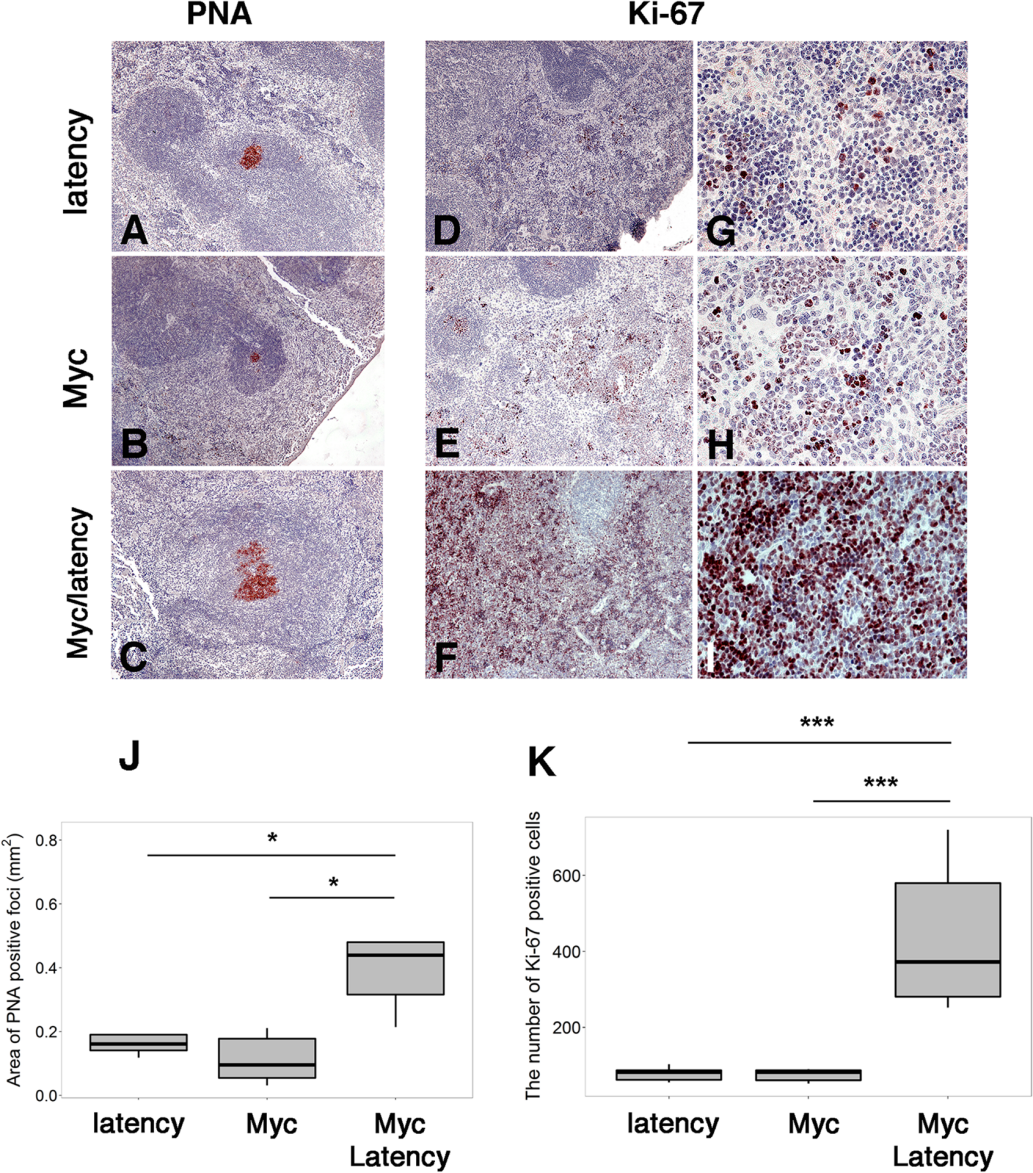
Augmented proliferation in Myc/latency mice. **(A-C)** PNA staining of spleen sections from the latency (n = 5) or the Myc (n = 5) or the Myc/ latency (n = 5). Ki-67 staining of spleen sections from the latency **(D, G**; n = 5) or the Myc **(E, H**; n = 5) or the Myc/ latency **(F, I**; n = 5). Representative images are shown. **(J)** The area of PNA-positive foci from panels A-C was plotted. **(K)** The number of Ki-67 positive cells from panels G-I was plotted. * and *** represent significant difference with p ≤ 0.05, and p ≤ 0.0005 by ANOVA, respectively.

Peanut agglutinin (PNA) is a known activation marker for the GC [26,29]. Enlarged PNA-positive patches in the GC of spleen is a phenotype of the KSHV latency locus [29], but not of this particular strain of Myc transgenic mice. PNA-positive foci were significantly larger in the Myc/latency double transgenic mice than those of either the latency or Myc mice (Fig 2A–2C). The area of PNA-positive foci in spleen was larger in the Myc/latency than in the Myc mice (Fig 2J). This data evidences KSHV-Myc cooperation in GC expansion.

The increased proliferative phenotype was confirmed using another clinically validated marker, Ki-67. The Myc/latency mice exhibited striking reactivity for Ki-67 in the red pulp region of spleen, where extramedullary hematopoiesis occurs in rodents. By contrast, the KSHV latency single transgenic and Myc single transgenic mice showed weak expression of Ki-67 (Fig 2D–2I). The difference in Ki-67 staining was significant to p ≤0.0004 by ANOVA (Fig 2K). The higher degree of Ki-67 positivity was verified in all mice per genotype (S4 Fig).

**Fig 3:**
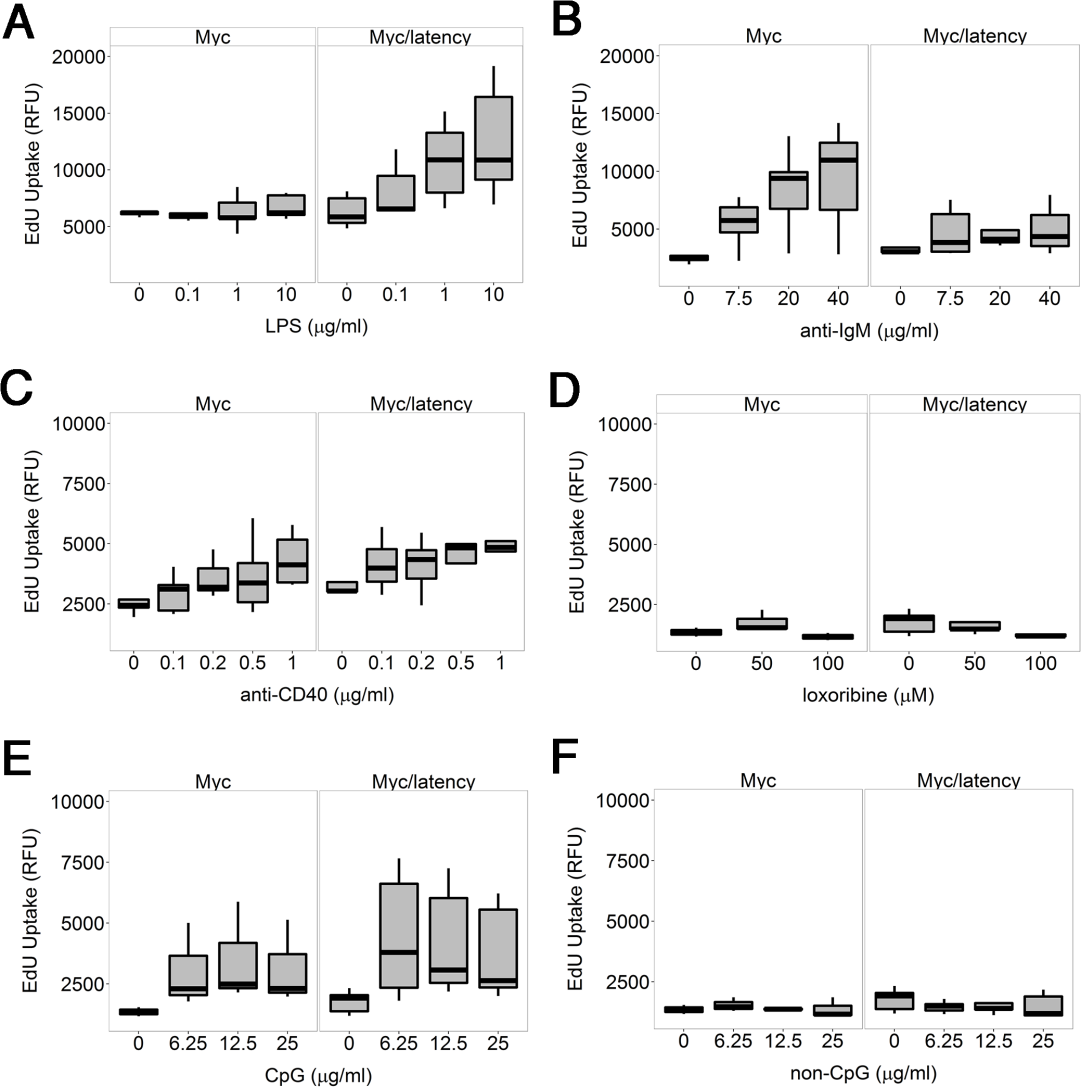
KSHV latency locus confers hyper-responsiveness to LPS in the environment of forced Myc overexpression. Proliferation was evaluated by incorporation of 5-ethynyl-2'-deoxyuridine (EdU) into DNA. Splenic B cells from the transgenic (n = 5) and wild-type mice (n = 5) were cultured with varying doses of LPS (A), or anti-IgM (B), or anti-CD40 antibody (C), or loxoribine (D), or CpG (E), or non-CpG (F) for 72 hours. Relative fluorescence unit (RFU) was measured and is expressed as ex vivo cell proliferation.

One hypothesis to explain how viral infection can facilitate B cell hyperplasia and lymphoma, is that the viral latent genes render infected B cells hyperresponsive to BCR and Toll-like receptor (TLR) signaling. We showed earlier that purified B cells from KSHV latency mice respond better to lipopolysaccharide (LPS), anti-IgM, and anti-CD40 [29]. As a polyvalent antigen, LPS activates both TLR and BCR signaling [35]. To test the hypothesis that the KSHV latency locus conferred a similar hyperresponsiveness in the Myc background, ex vivo proliferation of splenic B cells was assessed. Splenic CD19^+^ cells from the Myc/latency mice showed dose-dependent hyperresponsiveness to LPS, but no longer to anti-IgM or anti-CD40 or a TLR7 agonist, loxoribine or a TLR9 agonist, CpG-containing oligonucleotides (Fig 3). In the case of the LPS response, the difference between Myc and Myc/latency was significant to p ≤ 0.05 by ANOVA. The presence of the KSHV transgene increased the response to LPS. The presence of the KSHV transgene dampened the response to BCR crosslinking by anti-IgM antibody. This suggests that the KSHV latency locus augments TLR but not BCR-only or CD40L-only signaling pathways in the context of activated Myc.

### Lymphomagenesis in Myc/latency mice

The most stringent test for the presence of fully transformed B cells is the ex-vivo outgrowth assay in the absence of supportive growth factors. To examine the outgrowth potential of the Myc/latency mice, primary cells from spleen or BM in 9–11 week-old Myc (n = 6) or Myc/latency (n = 6) mice were seeded on methylcellulose media without B cell growth factors, and the number of colonies was counted. With the exception of one animal, splenocytes of the Myc/latency or the Myc mice did not produce colonies (S2 Table), though we routinely observed colonies from BM derived cells which were not significantly different between both genotypes (32.3 ± 14.3 for 6 Myc mice, 38.3 ± 9.0 for 6 Myc/latency mice; p ≤ 0.41).

**Fig 4:**
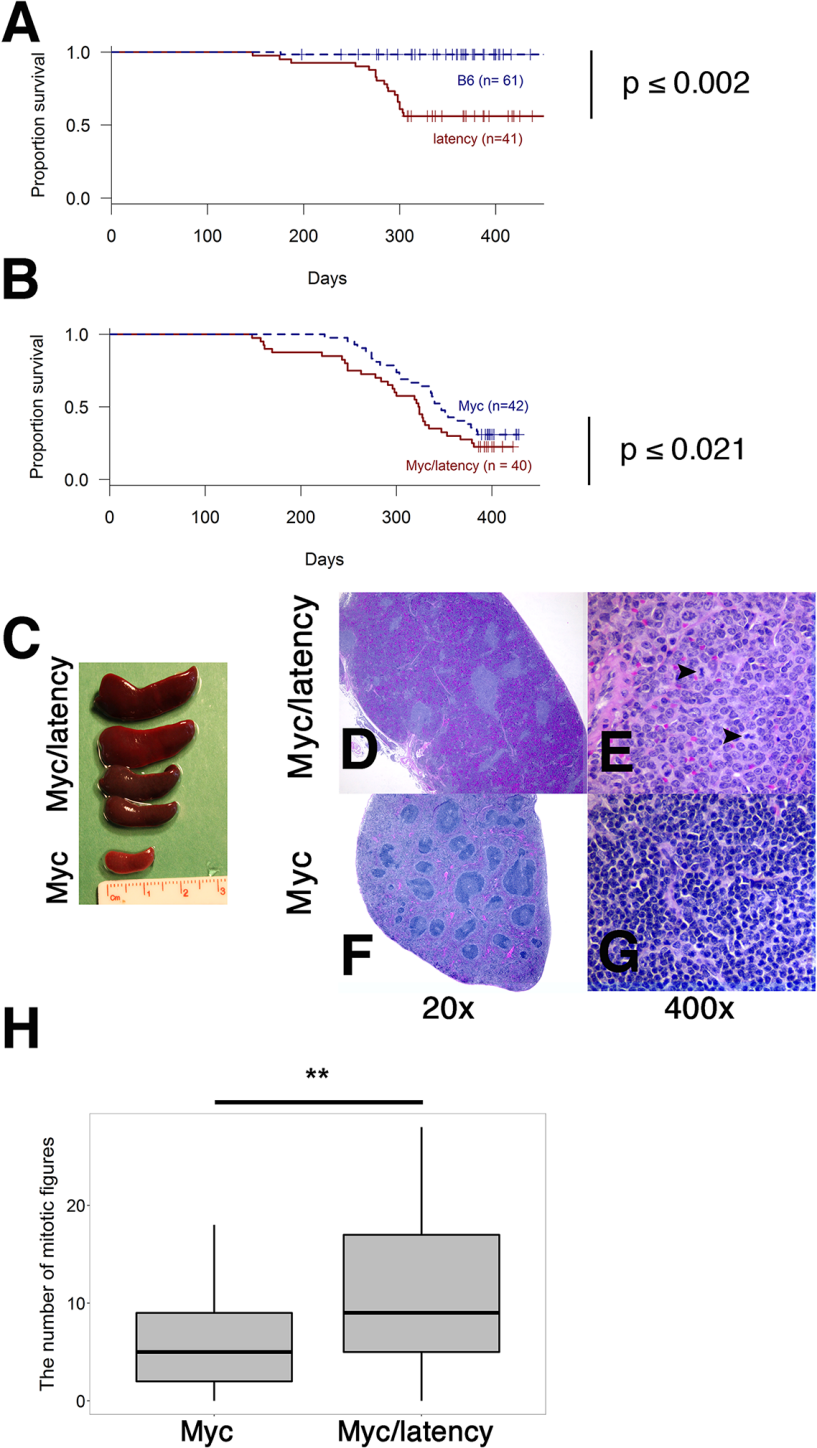
Augmented tumorigenicity by cooperation of KSHV latency locus and Myc. **(A-B)** Survival plot of the wild-type (C57BL/6) and latency, and the Myc and Myc/latency mouse cohorts. **(C)** Splenomegaly was observed in the Myc/latency mice. **(D-E)** Spleen section was shown and mitotic figures (black arrows) were found in the Myc/latency mouse. H&E staining. **(F-G)** Normal splenic architecture was presented in the Myc mouse. H&E staining. Representative images are shown. **(H)** Mitotic figures were counted for 5 high power field images (400X) per sample (42 for Myc and 40 for Myc/latency mice). ** represents significant difference with p ≤ 0.005 by ANOVA.

To formally test the hypothesis that Myc and KSHV latent genes cooperate to induce lymphoid hyperplasia and neoplasia, Myc transgenic (n = 42), the KSHV latency locus transgenic (n = 41), and Myc/KSHV latency locus double transgenic mice (n = 40) were monitored for 500 days (Fig 4A and 4B). Wild-type B6 mice were tumor-free for 500 days. Single transgenic Myc mice remained tumor-free until 200 days, while both latency and Myc/latency mice started to develop tumors around 130 days. The overall survival rate was significantly lower in the Myc/latency mice, when compared to that of Myc mice (p ≤ 0.021 by log-rank test) (Fig 4B). Given the weak tumor phenotypes of these particular Myc transgenic mice [7], we surmise that the increased rate of tumor incidence is attributable to cooperation of KSHV latent genes and Myc.

Pathological evaluation was performed on all mice. 11 (27.5%) and 20 (50.0%) out of 40 Myc/latency mice developed frank lymphoma and lymphoid hyperplasia in the spleen, respectively (Table 1). The lymphoma incidence rate of Myc/latency mice was marginally higher than that of the single KSHV transgenic mice, which was 17.1%, but significantly higher than in the Myc mice (4.8%, p ≤ 0.016 by F-test). Lymphoid hyperplasia can progress to frank lymphoma [36–38]. The combined rate for incidence of lymphoma and lymphoid hyperplasia was higher in Myc/latency mice (77.5%) than latency (69.1%) or Myc mice (43.9%). Examples of severe splenomegaly are shown in Fig 4C and an example of pathology for Myc/latency mice compared to normal spleen architecture in Fig 4D–4G. Mitotic figures were found in spleen from a mouse diagnosed as lymphocytic lymphoma (Myc/latency), while none were found in spleen diagnosed as lymphoid hyperplasia (Myc) (Fig 4E and 4G). The number of mitotic figures was significantly higher in the Myc/latency than the Myc mice (p ≤ 0.002 by ANOVA) (Fig 4H).

**Table 1:** Lymphoma development.

	Latency		Myc		Myc/latency			
	No. mice	rate (%)	No. mice	rate (%)	No. mice	rate (%)	P (vs Myc)*	P (vs Latency)*
lymphoma	7	17.1	2	4.8	11	27.5	0.016	0.035
lymphoid hyperplasia	11	26.8	27	64.3	20	50.0	1	0.005
normal	23	56.1	13	31.0	9	22.5		
total	41		42		40			
severe EMH	11	26.8	17	40.5	26	65.0	0.029	0.001

*: Data were analyzed using Fisher’s exact test. A p value ≤ 0.05 was regarded as significant.

**Fig 5:**
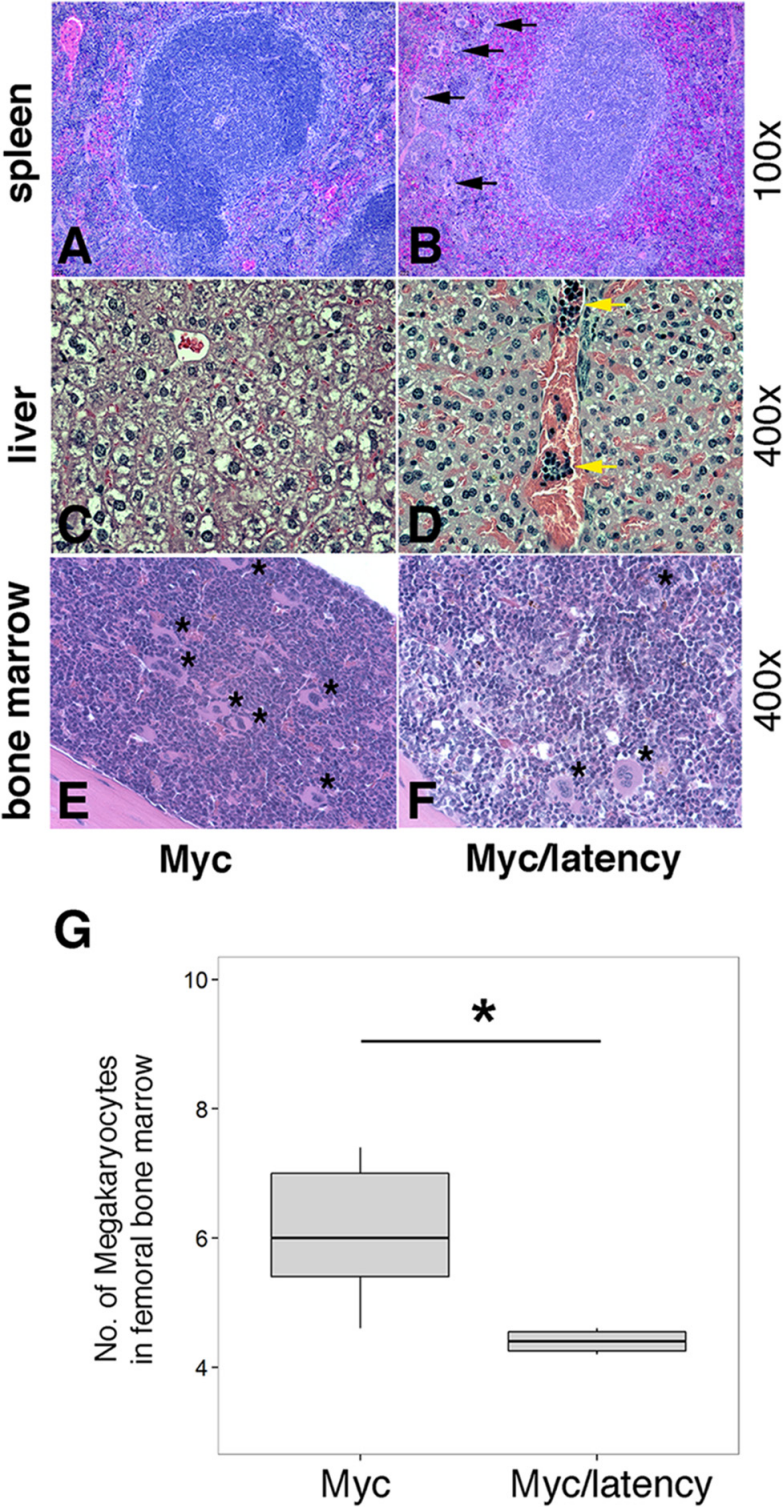
Severe EMH in the Myc/latency mice. **(A-B)** Severe EMH was found in spleen in the Myc/latency mice. Black arrow represents megakaryocyte. H&E staining. **(C-D)** Liver from the Myc/latency mice showed severe EMH. Yellow arrow indicates cluster of erythroid precursors in portal vein. H&E staining. **(E-F)** Decreased hematopoiesis was found in femoral BM from the Myc/latency mice. Black asterisk represents megakaryocyte. **(G)** The number of megakaryocytes was counted in 5 high field images (400X) per femoral BM section from 6 mice per each genotype and plotted. H&E staining. Representative images are shown.

Lymphoma observed in the Myc/latency mouse cohort is summarized in Table 2. Mice with early lymphoma or lymphocytic lymphoma exhibited disrupted splenic architecture and white pulp expanded by large lymphocytes with frequent mitotic figures, whereas mice diagnosed as normal had regular splenic architectures with clearly defined GCs. Mice with lymphoma also displayed severe extramedullary hematopoiesis, showing augmented frequency of megakaryocytes in spleen (Fig 5A and 5B) and elevated numbers of erythroid precursors in portal area of liver (Fig 5C and 5D). BM was examined to see if a failure in hematopoiesis from the Myc/latency mice may induce severe extramedullary hematopoiesis (EMH) in the spleen and liver for compensation (Fig 5E and 5F). Frequencies of myeloid and erythroid precursors were not significantly different between the Myc and the Myc/latency mice. However, the number of megakaryocytes was decreased in the Myc/latency mice (Fig 5G; p ≤ 0.017 by ANOVA), suggesting that inadequate hematopoiesis in BM from the Myc/latency mouse drives severe EMH in the spleen and liver. Mice diagnosed with lymphoid hyperplasia retained normal splenic follicular architecture, but lacked discernible GCs with pale MZ (Table 2; mouse #176). In sum, even the weak Cα Myc allele can cooperate with the KSHV latent locus to foster lymphoma development in vivo.

**Table 2:** Pathology on spleen from Myc/latency mice diagnosed with lymphoma.

Mouse #	Spleen	Length of spleen (cm)	Diagnosis
3	Disrupted normal architecture of spleen. No distinctive white pulp and red pulp. White pulp is expanded by large lymphocytes (blast type) with frequent mitotic figures.	2	lymphocytic lymphoma
170	White pulp nodules are composed of pale large lymphocytes and lack dark zone of small lymphocytes and discernible follicle structures. Severe splenic EMH	1.7	early lymphoma
176	Normal architectures are retained. White pulp nodules are composed of dark zone of small lymphocytes and pale marginal zone. No discernible germinal centers observed. Severe splenic EMH	1.8	Lymphoid hyperplasia*
192	White pulp nodules are composed of dark zone of small lymphocytes and pale marginal zone. No discernible germinal centers observed. Severe splenic EMH	1.8	early lymphoma
577	White pulp nodules are composed of pale large lymphocytes and lack dark zone of small lymphocytes and discernible follicle structures. Severe splenic EMH	3.2	lymphocytic lymphoma
591	Disrupted normal architecture of spleen. No distinctive white pulp and red pulp. White pulp is expanded by large lymphocytes (blast type) with frequent mitotic figures. Severe splenic EMH.	2.5	lymphocytic lymphoma
854	Normal architectures are retained. White pulp nodules are composed of dark zone of small lymphocytes and clear marginal zone. Clear germinal centers observed. Severe splenic EMH.	1.5	normal*
911	Disrupted normal architecture of spleen. No distinctive white pulp and red pulp. White pulp is expanded by large lymphocytes (blast type) with frequent mitotic figures. Severe splenic EMH.	3.3	lymphocytic lymphoma
939_02	Disrupted normal architecture of spleen. No distinctive white pulp and red pulp. White pulp is expanded by large lymphocytes (blast type) with frequent mitotic figures. Severe splenic EMH.	2.8	lymphocytic lymphoma

*: One case of lymphoid hyperplasia or normal was included.

## Discussion

Chromosomal translocation of Myc has been identified as the defining cellular driver mutation in BL [1]. Deregulated Myc activity is seen in the majority of DLBCL, though in PEL the myc locus appears to be normal [33,39]. Previous studies identified a number of chromosomal locations that were reproducibly amplified in PEL, such as FHIT and WWOX [40,41]; as well as activation of the BCR/PI3K and TLR/MyD88/IRAK signaling pathways [40,42,43]. Based on the biology of B cell lymphoma and the broad transcriptional phenotype of activated Myc, we hypothesized that deregulated Myc signaling can cooperate with BCR/TLR activation and KSHV latent genes to drive lymphomagenesis.

These experiments are not trivial, since the most penetrant Myc single-transgenic mice already exhibit a strong tendency towards multiple types of lymphoma. This made it difficult to detect cooperation of moderate human oncogenes. The one exception is BCL-2, which dramatically accelerates tumor development in the context of the Eμmyc mice [44]. BCL-2 is a broad-spectrum apoptosis suppressor, which counteracts the apoptosis signaling that emerges from many oncogenes, including heavily overexpressed Myc (reviewed in [1]). Myc is known to induce apoptosis by repressing the activity of Bcl-X_L_, an anti-apoptotic factor of BCL-2 family; mice expressing Myc and Bcl-X_L_ developed plasma cell tumors with a higher incidence rate and shorter onset time than single transgenic Myc mice [7].

By contrast to BCL-2, the KSHV latency locus seems to modulate B cell development more modestly with the aim of fine-tuning signals from exogenous antigens. Fine-tuning is the general modus operandi of miRNAs, including of viral miRNAs. The KSHV latency mice showed dramatically increased plasma cell frequency and an increased propensity to respond to TLR4 stimulation in vivo and in vitro [29]. Here we show that these phenotypes were maintained in the context of activated Myc. These new data informed our working model that the role of latent viral infection, EBV in the case of endemic BL (but not sporadic BL) and KSHV in the case of PEL and MCD, is to (i) increase the overall number of hyper proliferative cells in response to low-level, polyvalent antigen, and (ii) perhaps modulate their B cell fate towards immediate responder MZ B cells. This would provide a fertile “soil” or cellular environment into which additional host events, such as Myc pathway activation, can develop their fully transforming potential [45].

Expression of the KSHV latency genes in the context of activated Myc resulted in drastic plasmacytosis in the double transgenic mice. The PBs and PCs from spleen and BM in the latency mice were increased, while only splenic PBs were increased and PC development was dampened in the Myc mice [7,29,46]. The expansion of both PBs and PCs in the Myc/latency mice suggests that most of the PBs survived and differentiated terminally into PCs in spleen. However, the frequency of PCs was decreased in the BM, consistent with the idea that the some PCs failed to home to the BM after leaving the spleen or failed to survive in the BM. It is conceivable that the KSHV latency locus promotes the development of PBs into short-lived PCs in spleen, but not survival and/or homing of long-lived PCs to the BM. Understanding this aspect of KSHV biology is subject to further study.

Myc/latency compound transgenic mice developed lymphoma around 130 days with an incidence rate of 28%. In our colony, the iMycCα single-transgenic mice developed neoplasms at ~200 days with an incidence rate of 5%, which was slightly lower, but within the margin of error, than that observed in the original report (9%) [7]. KSHV latency single transgenic mice started to develop neoplasms at ~200 days, and the incidence rate was 17%, which was similar to our initial cohort (16%) [29]. This provides genetic evidence that the KSHV latency locus cooperates with Myc to drive B cell lymphoma.

The mechanism by which the KSHV latency locus cooperates with Myc to promote human PEL is not well understood. Structural deregulation of Myc is not common in PEL; rather various KSHV latent proteins have been proposed to deregulate Myc. Post-translational mechanisms typically lead to a lesser degree of oncogene activation than genomic translocation. LANA activates and stabilizes Myc in certain culture systems [31,39]. Myc also seems to be required for the maintenance of KSHV latency [47]. In cultured cells, mLANA, the murid herpesvirus-4 ortholog of KSHV LANA, stabilizes Myc through heterotypic polyubiquitination [48]. The KSHV vIRF-3/LANA2 also stimulates the transcription of Myc [32,49].

vFLIP cooperates with Eμ-driven Myc to promote lymphoma in double transgenic mice [50]. It also upregulates Myc, leading to protection of anti-IgM-induced apoptosis in the mouse indicator cell line, WEHI-231 [51]. The in vivo experiments reported here support these prior observations. Recent data suggest that the vFLIP protein is only very inefficiently expressed in natural infection of B cells [52], suggesting that even minute amounts of viral proteins have potent biological phenotypes. The current in vivo experiments reaffirm the ability of the KSHV latency locus to confer hyperresponsiveness to naïve B cells, which in the presence of elevated Myc activity leads to lymphoma. Most likely, KSHV latent genes act on multiple checkpoints along the pathway: initially by enhancing receptor-initiated signaling, and downstream of Myc, by ameliorating Myc’s tendency to induce apoptosis. Rather than dying, the activated plasmablasts continued to proliferate in the KSHV latency/Myc double transgenic mice (Fig 2).

One limitation of the current model is that it still lacks the contribution of the KSHV receptor homologs K1 and K15. Analogous to the EBV LMP-1 and LMP-2 proteins, these are believed to have an important role in modulating B cell biology [53–58]. In fact, the phenotypes seen here with only the nuclear KSHV latent genes are somewhat similar to early experiments using EBV nuclear latent proteins. The EBV^+^ eBL shows extremely restricted viral gene expression, i.e. only the EBV EBNA1 protein and the EBV miRNAs are detectable [59,60]. These seem, nevertheless, necessary for eBL cell survival [61]. By itself the EBV EBNA1 gene exhibits only weak phenotypes in vivo. It is associated with no, low, or late hyperplasia and lymphoma incidence, if driven by the IgH Eμ enhancer in transgenic mice [62–64]. EBNA-1 and Myc cooperate in inducing lymphoma [65]. LANA is the homolog of EBNA-1; it alone has only a minor growth modulating effect; is associated with low and late lymphoma incidence in transgenic mice [26,28]. Perhaps the missing factor in the initial LANA and EBNA-1 transgenic experiments was the absence of the viral miRNAs, which motivated us to use the complete KSHV latency model rather than the LANA single transgenic mice for our studies.

Taken together, this study reports that KSHV latency locus cooperates with Myc to promote lymphoma development in vivo. Compared to the low oncogenic potential of the iMycCα mice [7], this elevated tumorigenicity of the Myc/latency mice demonstrates pivotal roles of KSHV latency genes in viral lymphomagenesis in vivo.

## Materials and Methods

### Mice

Transgenic mice which express the KSHV latency locus were previously described [29]. Myc transgenic mice [C.129S1-*Igha* ^*tm1(Myc)Janz*^/J] were obtained from the Jackson Laboratory (Bar Harbor, ME) [7]. All mice were maintained under pathogen-free conditions using ventilated cages. All experiments were approved by the Institutional Animal Care and Use Committee (IACUC) at the University of North Carolina at Chapel Hill (UNC).

### Genotyping

Genomic DNA was isolated from mouse tail clipping using a Wizard SV genomic DNA kit (Promega). qPCR was performed for LANA and ApoB primers as previously described [28]. Mice with overexpressed Myc were typed by PCR according to supplier’s protocol using primers oIMR8447 & oIMR8448 for wild-type mice and oIMR8450 & oIMR8453 for the Myc mice (http://jaxmice.jax.org/protocolsdb/f?p=116:2:3011848657952163::NO:2:P2_MASTER_PROTOCOL_ID,P2_JRS_CODE:5234,008341).

### Pathology evaluation

Gross pathology evaluation and tissue extraction were done at the time of euthanization or death due to serious illness. Pathological diagnosis, including lymphoma and other types of malignancies, was done by a veterinary pathologist (Y. Kim) based on H&E staining and the morphological and histological aberrations observed in spleen, liver and bone marrow. Myeloid, erythroid precursors, and megakaryocytes were also evaluated on the all tissues. All pathological evaluation was performed using a microscope (Nikon ECLIPSE Ci Y-TV55, Japan). Images were captured using a camera (Jenoptik ProgRes SpeedXT core 3, Germany), and acquired using ProgRes CapturePro (Version 2.8, Jenoptik). The magnifications of the objective lenses were x2 or x10 or x40.

### Antibodies

The following antibodies were used for flow cytometry and immunohistochemistry. Polyclonal anti-mouse CD3 (Abcam); Fluorescein isothiocyanate (FITC)-conjugated anti-mouse IgD (clone 11-26c.2a), phycoerythrin (PE)-conjugated anti-mouse CD138 (clone 281–2), anti-mouse CD21/CD35 (clone 7G6), and anti-mouse IgM (clone R6-60.2) (BD Biosciences); biotin-conjugated anti-mouse ki-67 (clone SP6; Fisher); allophycocyanin (APC)-conjugated anti-mouse CD19 (clone 6D5), anti-mouse CD23 (clone B3B4), and FITC-conjugated anti-mouse CD45R (clone RA3-6B2) (Invitrogen); goat polyclonal anti-mouse IgG, and biotinylated anti-mouse CD45R (clone RA3-6B2) (Southern Biotech); biotinylated peanut agglutinin (PNA) (Vector Laboratories).

### Histology and immunohistochemistry

All tissues were extracted at the time of euthanization or death due to serious illness and were paraffin embedded and sectioned at the Animal Histopathology Core facility of UNC Lineberger Comprehensive Cancer Center (LCCC). Sections were stained with H&E at the same facility. Immunohistochemistry was performed as previously described [29]. Formalin-fixed paraffin-embedded spleen sections were incubated with PNA (1:200 dilution), anti-mouse Ki-67 (1:200 dilution), or anti-mouse IgG (1:100 dilution). The area of PNA-positive foci was measured using ImageJ [66]. The staining was visualized using a microscope (Leica DMLS, Germany) with the magnifications of the objective lenses of x4 or x10 or x40. Images were captured using a camera (Leica DFC480) and acquired using FireCam (Version 3.0, Leica). Staining intensity and prevalence was evaluated as previously described [67].

### Flow cytometry

Flow cytometry was performed as previously described [28]. Briefly, single cells were isolated from the spleen or bone marrow in 7–11 week-old Myc or Myc/latency mice. After red blood cell lysis, one million cells were subject to staining and flow analysis. Data acquisition was performed using a CyAn instrument (Beckman Coulter) at the UNC Flow core and the analysis was done using Flowjo Ver. 7.6.5 (Tree Star).

### B-cell isolation and proliferation

Splenic B cells were purified from 11–13 week-old Myc (n = 5) or Myc/latency (n = 5) mice using an EasySep Mouse B Cell Enrichment Kit (StemCell Technologies). B cells were cultured in RPMI 1640 medium supplemented with 20% fetal bovine serum, 2 mM L-glutamine, penicillin (0.05 μg/mL), and streptomycin (5 U/mL) (Invitrogen) with CD40 mAb (clone HM40-3, Biolegend), F(ab′)2 goat anti-mouse IgM Ab (Jackson ImmnuoResearch Laboratory), LPS (from *Escherichia coli* 0111:B4), loxoribine, or Class B CpG oligonucleotide (Invivogen) at 37°C under 5% CO_2_. The ex vivo cell proliferation rate was determined using a Click-iT EdU microplate assay kit (Invitrogen) according to the supplier’s protocol. The incorporated EdU in DNA was conjugated with Oregon Green-azide, and coupled with horseradish peroxidase-labeled anti-Oregon Green antibody. The relative fluorescence unit (RFU) at 485 nm excitation/585 nm emission was measured using a Fluostar Optima instrument (BMG, Inc.), and expressed as the ex vivo proliferation rate of the B cells.

### Colony-forming cell assay

Ten million splenic B cells from each mouse (7–11 weeks old) were cultured on semisolid methylcellulose media (M3134 from StemCell Technologies) supplemented with 20% fetal bovine serum, 2 mM L-glutamine, penicillin (0.05 μg/mL), streptomycin (5 U/mL), 7.5% sodium bicarbonate, and 55 mM 2-mercaptoethanol (all from Invitrogen). The number of colony-forming cells (CFC) was counted on 14 days after culture. One half million bone marrow (BM) cells from each mouse (7–11 weeks old) were cultured on semisolid media (M3630 from StemCell Technologies) and the number of CFC was counted on 9 days after culture.

### IgG isotyping by ELISA

Serum was collected from both the Myc and the Myc/latency mice (7–10 weeks old). Igs were measured as previously described [29].

### Statistical analysis

Data in figures and text were represented as mean ± standard deviation. Continuous data were analyzed using ANOVA and adjusted for multiple comparisons by Dunnett method using R version 3.1.1 (2014-07-10). Incidence data were analyzed using Student’s t-test or Fisher’s exact test. A p value ≤ 0.05 was regarded as significant. For box plots, the box represents the interquartile range and the line within the box represents the median. The lower limit of a lower vertical segment points 5% percentile and the upper limit of an upper vertical segment is 95% percentile.

### Ethics statement

All animal work was approved by the IACUC committee of the University of North Carolina at Chapel Hill under #13–219.0/KSHV latency mice. All work has been conducted in accordance with the Public Health Service (PHS) policy on Humane Care and Use of Laboratory Animals, the Amended Animal Welfare Act of 1985, and the regulations of the United States Department of Agriculture (USDA).

## Supporting Information

S1 Table B cell populations in the Myc and Myc/latency mice. (DOCX)

S2 Table The number of colonies found in methylcellulose culture by splenocytes from the Myc or Myc/latency mice (n = 12). (DOCX)

S1 Fig Genotyping of the Myc mice. Primer pair (oIMR8447 and oIMR8448) was used for genotyping wild-type mice (lanes 5 and 7; 1300 bp) and oIMR8450 and oIMR8453 for the Myc mice (lanes1-4, 6; 700 bp). MW represents a molecular marker.
(TIF)

S2 Fig Transcription of KSHV latent genes and miRNAs. Total RNA from splenocytes were analyzed using RT-qPCR. GAPDH was used as a positive control and NTC means non-template control.
(TIF)

S3 Fig Consistent splenic plasmacytosis in the Myc/latency mice. Higher staining intensity and prevalence of anti-mouse γ-chain was observed in spleen sections of all Myc/latency mice (n = 40) than the Myc mice (n = 42). Representative images are shown. Magnification X400. Igγ-chain positive cells were counted in spleen section from 42 Myc and 40 Myc/latency mice and plotted. * represent significant difference with p ≤ 0.05 by ANOVA.
(TIF)

S4 Fig Augmented proliferation was consistent in the Myc/latency spleen. Ki-67 was used to assess proliferation in spleen from all Myc (n = 42) and Myc/latency (n = 40) mice. Representative images are shown. Magnification X400. Ki-67 positive cells were counted in spleen section from 42 Myc and 40 Myc/latency mice and plotted. *** represent significant difference with p ≤ 0.0005 by ANOVA.
(TIF)
