# Supplementary material for: KSHV Latency Locus Cooperates with Myc to Drive Lymphoma in Mice
Source: PLoS Pathog. 2015 Sep 1;11(9):e1005135. doi: 10.1371/journal.ppat.1005135 (PMC4556645; doi:10.1371/journal.ppat.1005135)
Supplement: S1 Table — (DOCX) [file ppat.1005135.s001.docx]

**Supplementary Table 1**

B cell populations in the Myc and Myc/latency mice

|  |  |  | | |  |  | | |  |
| --- | --- | --- | --- | --- | --- | --- | --- | --- | --- |
|  |  | **Frequency** | | | | | | |  |
|  |  | **Myc** | | |  | **Myc/latency** | | |  |
| **Class** | **Marker** | **%** | **SD** | **n** |  | **%** | **SD** | **n** | **P** |
| Mature | CD19+IgM+IgD+ | 32.18 | 14.70 | 5 |  | 32.90 | 9.05 | 5 | NS |
| Activated B | CD19+IgM+IgD+FSC-hi | 4.76 | 0.68 | 5 |  | 3.24 | 0.98 | 5 | NS |
| MZ | CD19+IgM+IgD- | 25.32 | 5.42 | 5 |  | 24.08 | 12.60 | 5 | NS |
| Activated MZ | CD19+IgM+IgD-FSC-hi | 9.66 | 1.59 | 5 |  | 10.94 | 8.86 | 5 | NS |
| MZ | CD19+CD21hiCD23- | 11.52 | 4.17 | 5 |  | 10.33 | 2.12 | 5 | NS |
| FO | CD19+CD21-CD23+ | 55.04 | 13.70 | 5 |  | 68.90 | 13.47 | 5 | NS |
| Transitional | CD19+CD21-CD23- | 19.82 | 10.98 | 5 |  | 12.30 | 10.16 | 5 | NS |
| Plasmablasts  (spleen) | CD19-B220+CD138+ | 0.31 | 0.10 | 5 |  | 0.50 | 0.10 | 5 | 0.03 |
| Plasma cells (Spleen) | CD19-B220-CD138+ | 0.21 | 0.05 | 5 |  | 0.55 | 0.21 | 5 | 0.04 |
| Plasmablasts  (Bone marrow) | CD19-B220+CD138+ | 0.15 | 0.08 | 5 |  | 0.09 | 0.03 | 5 | NS |
| Plasma cells (Bone marrow) | CD19-B220-CD138+ | 0.31 | 0.07 | 5 |  | 0.09 | 0.03 | 5 | 0.001 |

MZ, marginal zone B cells; FO, follicular B cells; SD, Standard Deviation; n, number of animal analyzed; NS, not significant
