# Supplementary material for: KSHV Latency Locus Cooperates with Myc to Drive Lymphoma in Mice
Source: PLoS Pathog. 2015 Sep 1;11(9):e1005135. doi: 10.1371/journal.ppat.1005135 (PMC4556645; doi:10.1371/journal.ppat.1005135)
Supplement: S2 Table — (DOCX) [file ppat.1005135.s002.docx]

Supplementary Table 2

The number of colonies found in methylcellulose culture by splenocytes from the Myc or Myc/latency mice (n = 12)

| Myc | Myc/latency |
| --- | --- |
| 0 | 0 |
| 0 | 0 |
| 0 | 10 |
| 0 | 0 |
| 0 | 0 |
| 0 | 0 |
| 0 | 0 |
| 0 | 0 |
| 0 | 0 |
| 0 | 0 |
| 0 | 0 |
| 0 | 0 |
