# Supplementary figures and images for: KSHV Latency Locus Cooperates with Myc to Drive Lymphoma in Mice
Source: PLoS Pathog. 2015 Sep 1;11(9):e1005135. doi: 10.1371/journal.ppat.1005135 (PMC4556645; doi:10.1371/journal.ppat.1005135)

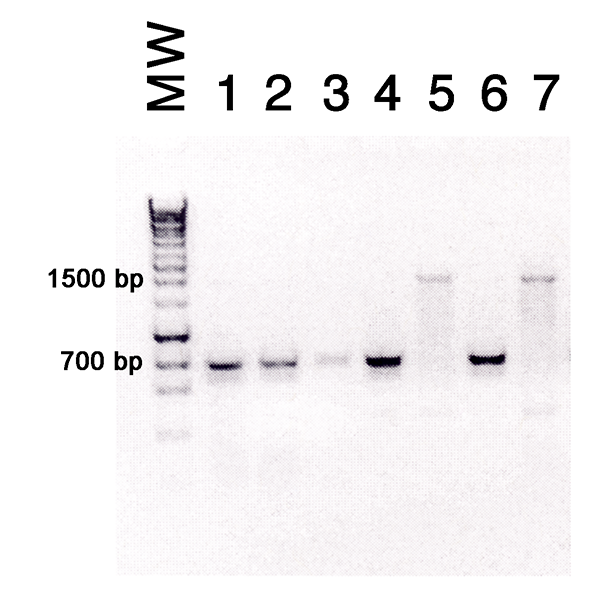

Supplement: S1 Fig — Primer pair (oIMR8447 and oIMR8448) was used for genotyping wild-type mice (lanes 5 and 7; 1300 bp) and oIMR8450 and oIMR8453 for the Myc mice (lanes1-4, 6; 700 bp). MW represents a molecular marker. (TIF) [file ppat.1005135.s003.tif]

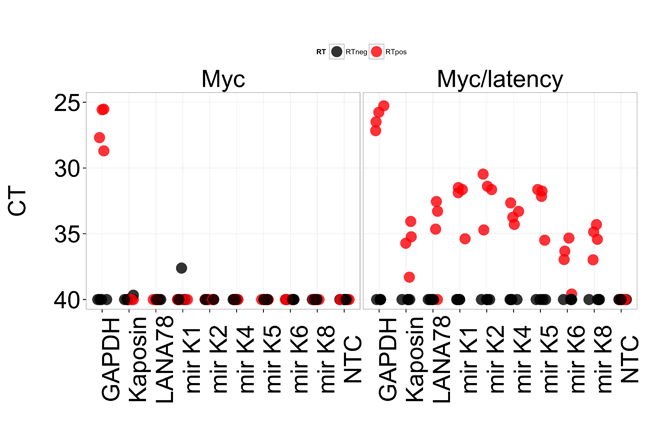

Supplement: S2 Fig — Total RNA from splenocytes were analyzed using RT-qPCR. GAPDH was used as a positive control and NTC means non-template control. (TIF) [file ppat.1005135.s004.tif]

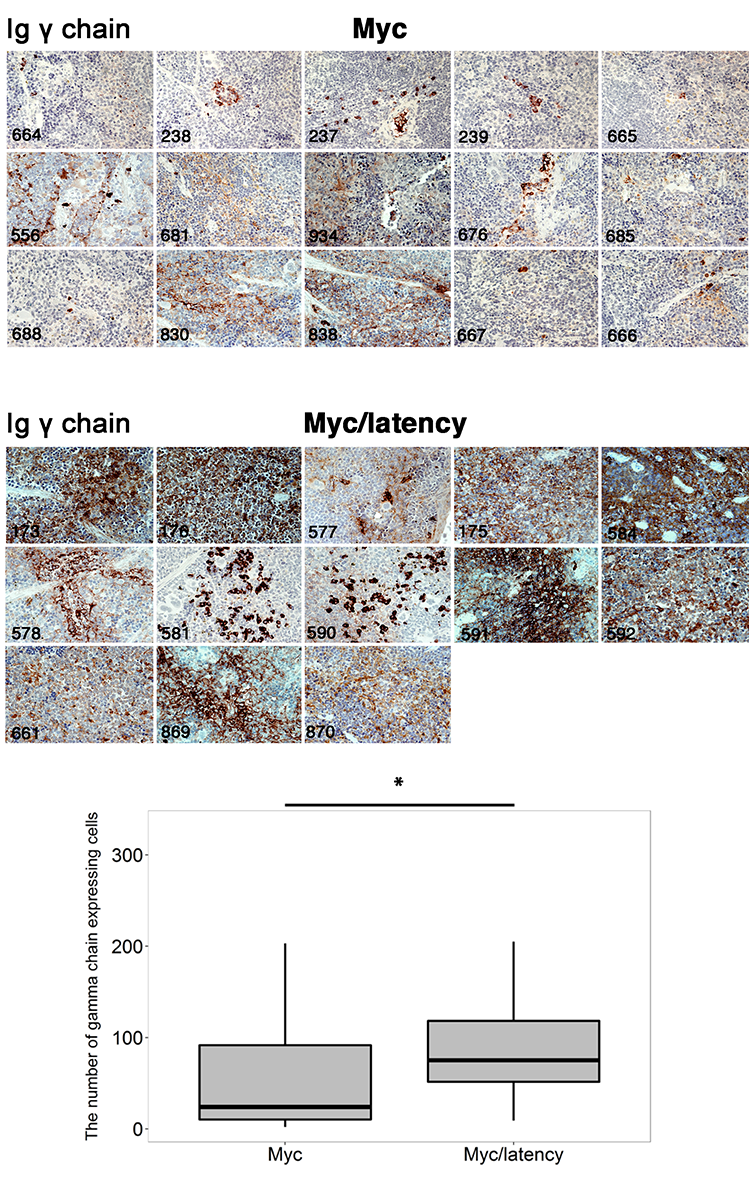

Supplement: S3 Fig — Higher staining intensity and prevalence of anti-mouse γ-chain was observed in spleen sections of all Myc/latency mice (n = 40) than the Myc mice (n = 42). Representative images are shown. Magnification X400. Igγ-chain positive cells were counted in spleen section from 42 Myc and 40 Myc/latency mice and plotted. * represent significant difference with p ≤ 0.05 by ANOVA. (TIF) [file ppat.1005135.s005.tif]

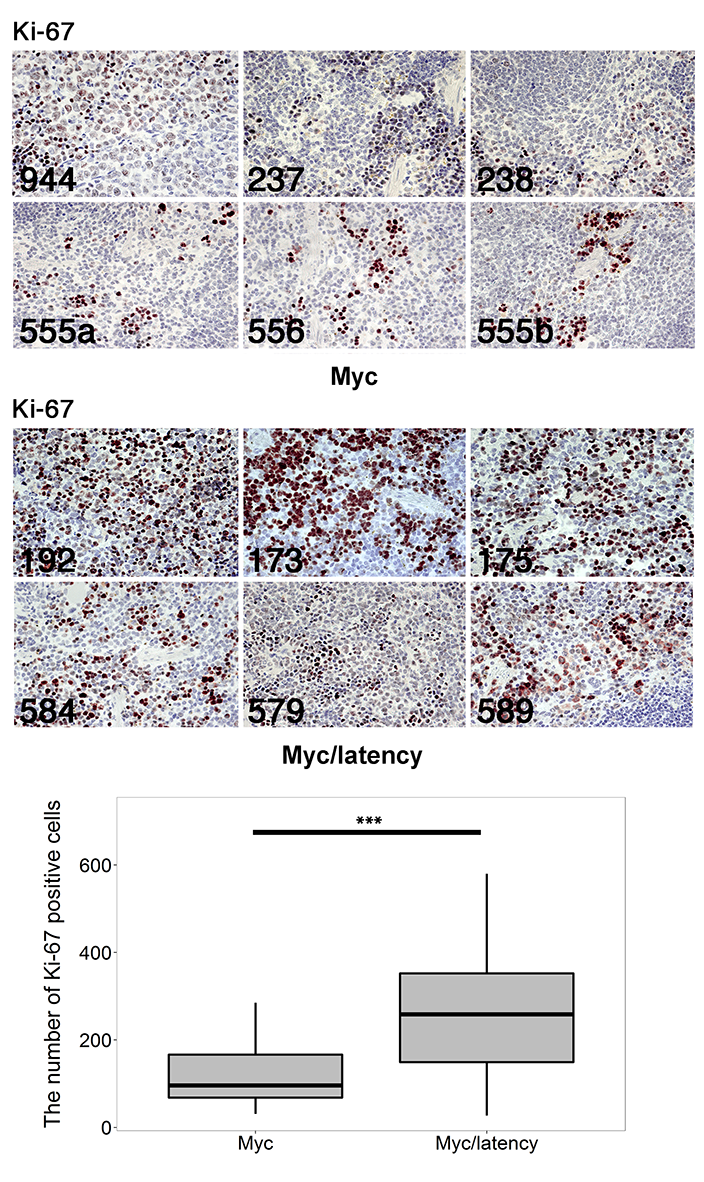

Supplement: S4 Fig — Ki-67 was used to assess proliferation in spleen from all Myc (n = 42) and Myc/latency (n = 40) mice. Representative images are shown. Magnification X400. Ki-67 positive cells were counted in spleen section from 42 Myc and 40 Myc/latency mice and plotted. *** represent significant difference with p ≤ 0.0005 by ANOVA. (TIF) [file ppat.1005135.s006.tif]
